# Acting on Hormone Receptors with Minimal Side Effect on Cell Proliferation: A Timely Challenge Illustrated with GLP-1R and GPER

**DOI:** 10.3389/fendo.2013.00050

**Published:** 2013-04-29

**Authors:** Véronique Gigoux, Daniel Fourmy

**Affiliations:** ^1^Université de Toulouse, Université Paul SabatierToulouse, France

**Keywords:** receptor, G protein, arrestin, GLP-1, estrogen, biased ligands, adverse effects, cancer

## Abstract

G protein-coupled receptors (GPCRs) constitute a large family of receptors that sense molecules outside the cell and activate inside signal transduction pathways and cellular responses. GPCR are involved in a wide variety of physiological processes, including in the neuroendocrine system. GPCR are also involved in many diseases and are the target of 30% of marketed medicinal drugs. Whereas the majority of the GPCR-targeting drugs have proved their therapeutic benefit, some of them were associated with undesired effects. We develop two examples of used drugs whose therapeutic benefits are tarnished by carcinogenesis risks. The chronic administration of glucagon-like peptide-1 (GLP-1) analogs widely used to treat type-2 diabetes was associated with an increased risk of pancreatic or thyroid cancers. The long-term treatment with the estrogen antagonist tamoxifen, developed to target breast cancer overexpressing estrogen receptors ER, presents agonist activity on the G protein-coupled estrogen receptor which is associated with an increased incidence of endometrial cancer and breast cancer resistance to hormonotherapy. We point out and discuss the need of pharmacological studies to understand and overcome the undesired effects associated with the chronic administration of GPCR ligands. In fact, biological effects triggered by GPCR often result from the activation of multiple intracellular signaling pathways. Deciphering which signaling networks are engaged following GPCR activation appears to be primordial to unveil their contribution in the physiological and physiopathological processes. The development of biased agonists to elucidate the role of the different signaling mechanisms mediated by GPCR activation will allow the generation of new therapeutic agents with improved efficacy and reduced side effects. In this regard, the identification of GLP-1R biased ligands promoting insulin secretion without inducing pro-tumoral effects would offer therapeutic benefit.

## Introduction

Seven transmembrane receptors, also termed G protein-coupled receptors (GPCR), form the largest class of the cell surface membrane receptors, involving 850 members in the human genome. GPCR are generally expressed in several different tissues in the same individual and involved in numerous physiological processes, including in the neuroendocrine system by playing a pivotal role in the control of feeding behavior, reproduction, growth, hydromineral homeostasis and stress response. At the cellular level, biological effects triggered by GPCR often result from the activation of multiple intracellular signaling pathways which are dependent or independent of G protein coupling (Rajagopal et al., 2010a).

Near 30% of therapeutic agents on the pharmaceutical market target GPCR (Hopkins and Groom, 2002). Whereas the majority of the GPCR-targeting drugs have proved their therapeutic benefit, some of them were associated with undesired effects (Table 1). Around 70% of the drugs which target GPCR are derived from the natural ligand and the use of agonist mimetics in clinical indication can act on the different tissues expressing the targeted GPCR and potentially induce undesired effects. Notably, prolonged treatment with GPCR-targeting agonist analogs was shown to induce preneoplastic and tumoral side effects. Here, we develop two examples of the use of GPCR-targeting drugs whose therapeutic benefits are tarnished by carcinogenesis risks. As a first example, glucagon-like peptide-1 receptor (GLP-1R) agonists used as anti-diabetic treatment were shown to induce preneoplastic lesions and/or cancers in the pancreas and the thyroid. The second example of ligands that we chose to develop does not initially target a GPCR, but the unexpected undesired effect is associated with a new GPCR target. Indeed, nuclear estrogen receptor antagonists such as tamoxifen are a breakthrough in the therapy and the prevention of breast cancer; however, long-term treatment was shown to be associated with an increased risk in endometrial cancer which was explained by the tamoxifen-induced activation of a GPCR, named G protein-coupled estrogen receptor (GPER).

**Table 1:** **Examples of ligands used for clinical indication in endocrinology with undesired side effect**.

Receptor	Ligand	Clinical indication	Undesired side effects	Reference
Dopamine-R	Antagonist	Schizophrenia/bipolar disorder (*Central nervous system*)	Diabetes (*Pancreatic β-cells:serotonin, histamine, muscarinic antagonism*)	Nasrallah (2008), Medved et al. (2009)
EstrogenR/GPER	Tamoxifen	Breast cancer/osteoporosis	Endometrial cancer/uterine sarcoma/ovarian cancer Tamoxifen breast cancer resistance	Du et al. (2012a), Ignatov et al. (2010a), He et al. (2012) Wei et al. (2012)
GLP-1R	GLP-1 analogs	Diabetes (*pancreatic* β *cell*)	Preneoplasia/pancreatitis (*Pancretic duct cell*) Medullary thyroid cancer (*Thyroid C-cell*)	Nachnani et al. (2010), Gier et al. (2012a), Elashoff et al. (2011) Bjerre Knudsen et al. (2010), Madsen et al. (2012), Victoza (Liraglutide) Injection (2012)
GnRH-R	Agonist	Prostate cancer (*anterior pituitary*)	Diabetes	Kintzel et al. (2008), Saylor and Smith (2009)
NPY-R		Eating disorders (*Brain*)	Neuroblastoma	Lu et al. (2010)
PTH-R	Agonist	Osteoporosis (*Osteoblast*)	Osteosarcoma (*Mesenchymal stem cell*)	Subbiah et al. (2010), Hodsman et al. (2005)
Serotonin 5HT4R	Agonist	Gastrointestinal disorder (*enteric nervous system in GI tract*)	Cardiovascular disease	Tack et al. (2012)
SST-R	Somatostatin analogs	Acromegaly (*Pituitary: suppress GH/IGF-1 secretion*) Carcinoid tumors/Vipomas Endocrine tumor	Hypo/hyperglycemia Hypothyroidism (Pituitary: suppress secretion TSH) Pancreatitis	

*The cells or organs targeted by the drug in the clinical indication or in the adverse effects are given in parenthesis*.

We point out and discuss the need of more pharmacological studies to understand and overcome the undesired effects associated with the chronic administration of ligands which target GPCR. Deciphering the signaling networks engaged following GPCR activation appears to be primordial to unveil their contribution in the physiological and physiopathological processes.

## The Glucagon-Like Peptide-1 Receptor

**Figure 1:**
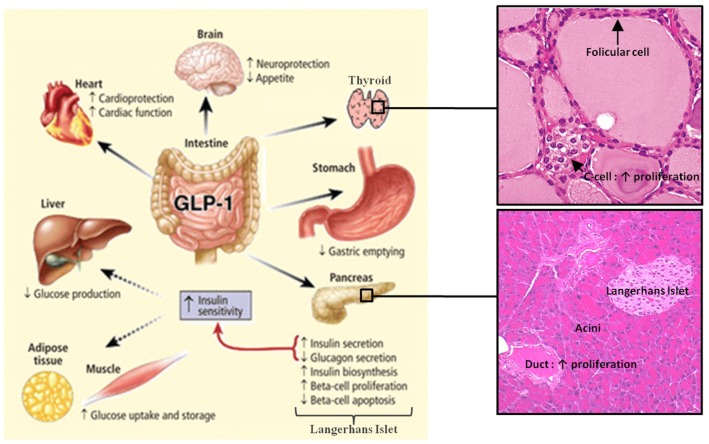
**Actions of GLP-1 in peripheral tissues**. Most of the effects of GLP-1 are mediated by direct interaction with GLP-1R on specific tissues. However, the actions of GLP-1 in liver, fat, and muscle most likely occur through indirect mechanisms. GLP-1 induces the proliferation of pancreatic duct cells and thyroid C-cells. Reprinted from Gastroenterology (Baggio and Drucker, 2007).

**Figure 2:**
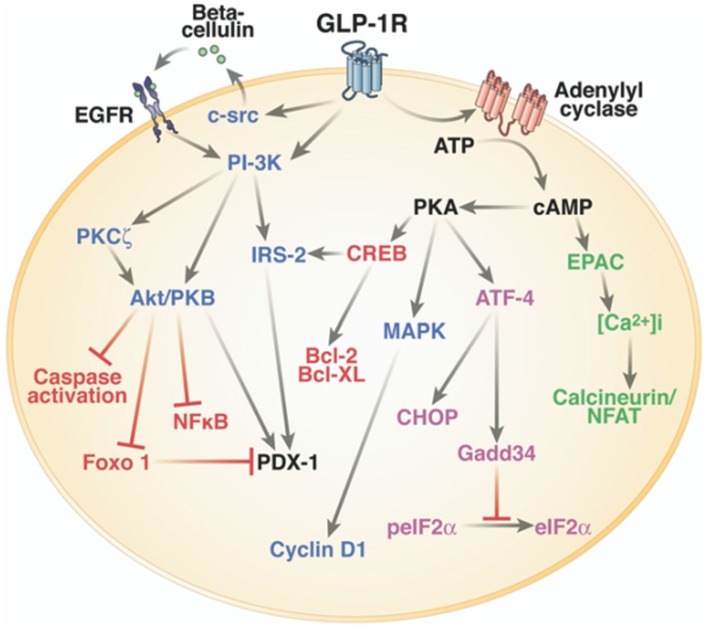
**Intracellular signaling pathways of GLP-1R in the pancreatic β-cell**. One of the main physiological roles of GLP-1 is to enhance insulin secretion in a glucose-dependent manner. To stimulate insulin secretion and biosynthesis (green), GLP-1R coupled to adenylyl cyclase leading to the activation of cAMP-regulated guanine nucleotide exchange factor II (cAMP-GEFII, also known as Epac2) signaling pathway. GLP-1 plays also a key role in the homeostasis of β-cell mass by inducing β-cell proliferation (blue) and protecting against apoptosis (red). These functions are mediated *via* the activation of the cAMP/PKA/CREB (cAMP-responsive element binding protein) and the transactivation of the epidermal growth factor receptor (EGF-R) leading to the activation of phosphatidylinositol-3 kinase (PI3K), Protein Kinase Cζ (PKCζ), Akt-protein kinase B, ERK1/2 (Extracellular Regulated Kinase, named also MAPK, Mitogen-Activated Protein Kinase) signaling pathways, and to the up-regulation of the expression of the cell cycle regulator cyclin D1. GLP-1R agonists also improve β-cell function and survival during endoplasmic reticulum stress (purple) by enhancing of ATF-4 translation in a cAMP- and PKA-dependent manner, promoting the up-regulation of the endoplasmic reticulum stress markers CHOP and GADD34 expression and the dephosphorylation of eIF2α. Of note, there is considerable overlap between pathways induced by the GLP-1R activation. Reprinted from Gastroenterology (Baggio and Drucker, 2007).

One of the main physiological roles of GLP-1 is to enhance insulin secretion in a glucose-dependent manner. Thus, GLP-1 is an incretin hormone released after meals by L cells in the intestine (Figure 1) (Mojsov et al., 1987). GLP-1 exerts its physiological effects through binding to its specific G protein-coupled receptor, GLP-1R, which is primarily and positively coupled to adenylate cyclase, through Gαs-containing heterotrimeric G proteins, leading to the activation of second messenger pathways such as protein kinase A (PKA) and cAMP-regulated guanine nucleotide exchange factor II (cAMP-GEFII, also known as Epac2) signaling pathways (Figure 2) (Thorens, 1992; Kashima et al., 2001; Mayo et al., 2003; Holz, 2004; Seino and Shibasaki, 2005; Doyle and Egan, 2007; Holst, 2007). In addition to its stimulatory effect on insulin secretion, GLP-1 suppresses the secretion of glucagon, a counter-hormone to insulin, thus maintaining glucose homeostasis following a meal (Nauck et al., 1993). GLP-1 plays also a key role in the homeostasis of β-cell mass by inducing β-cell proliferation and protecting against apoptosis which favor an expansion of β-cell mass (Figure 2) (Doyle and Egan, 2007). These functions are mediated *via* the activation of the cAMP/PKA/CREB (cAMP-responsive element binding protein) and the transactivation of the EGF-R (epidermal growth factor receptor) leading to the activation of phosphatidylinositol-3 kinase (PI3K), Protein Kinase Cζ (PKCζ), Akt-protein kinase B, Extracellular Regulated Kinase (ERK1/2) signaling pathways and to the up-regulation of the expression of the cell cycle regulator cyclin D1 (Buteau et al., 2003; Drucker, 2003; Trumper et al., 2005; Park et al., 2006; Doyle and Egan, 2007). The antiapoptotic effect of GLP-1 in β-cells also involves β-arrestin1 recruitment by GLP-1R which mediates the ERK1/2 activation leading to the phosphorylation and inactivation of the pro-apoptotic protein Bad (Quoyer et al., 2010). The properties of GLP-1 on insulin secretion and β-cell proliferation make GLP-1 one of the most promising therapeutic agent to treat type-2 diabetes. Moreover, GLP-1 analogs offer the advantage of improved glycemic control of type-2 diabetic patients, without inducing severe hypoglycemia (Phillips and Prins, 2011).

On the other hand, GLP-1 receptor activation directly promotes cell proliferation and enhances cell survival in several tissues including neurons, fibroblasts, and cardiomyocytes (Brubaker and Drucker, 2004).

### Could anti-diabetic treatment with GLP-1 analogs induce cancers?

Two GLP-1 mimetic drugs are now widely used to treat type-2 diabetes, exendin-4/exenatide and liraglutide, because of their optimal glucose lowering capacity with low risk of hypoglycemia (Chia and Egan, 2008; Buse et al., 2009; Nauck et al., 2009). Preclinical and clinical studies indicated that exenatide and liraglutide exert a positive effect on insulin secretion, β-cell proliferation, and survival (Goke et al., 1993; Chang et al., 2003; Drucker, 2006; Vilsboll et al., 2007, 2008; Pratley and Gilbert, 2008; Madsbad, 2009; Vilsboll, 2009). On the other hand, recent studies showed that the use of these GLP-1R agonists in anti-diabetic treatment can be associated with an increase of cancer risk. The main organs where concerns exist about the trophic effects of GLP-1 analogs and their potential carcinogenic propensity are the pancreas and the thyroid, both organs expressing GLP-1R.

#### The pancreas

Recent studies reported that both treatments with exenatide and liraglutide are associated with an increased risk of pancreatitis in humans, a disease which represents a known risk factor for pancreatic cancer (Denker and Dimarco, 2006; Cure et al., 2008; Tripathy et al., 2008; Greer and Whitcomb, 2009). The chronic administration of GLP-1 agonists was also shown to be associated with increased serum lipase and amylase in many patients with type-2 diabetes, suggesting pancreatic damage and inflammation (Lando et al., 2012). Evaluation of the U.S. Food and Drug Administration (FDA) adverse events database by Elashoff et al. (2011), showed 10- and 3-fold increases in the incidence of pancreatitis and pancreatic cancer, respectively, in diabetic patients treated with exenatide as compared to other therapies (rosiglitazone, nateglinide, repaglinide, and glipizide) (Elashoff et al., 2011).

Undesired effects were also observed on different animal models. Indeed, chronic administration of exenatide during 12 weeks increased pancreatic acinar inflammation, sensitized to pancreatitis, and promoted pancreatic duct hyperplasia in rats or in the LSL-Kras^G12D/+^/Pdx1-Cre^±^ murine model of pancreatic carcinogenesis (Nachnani et al., 2010; Gier et al., 2012a). The authors of this study related these adverse effects to the expression of GLP-1R in duct cells of the exocrine pancreatic tissue (Gier et al., 2012a). Whereas GLP-1R expression is clearly established in normal β-cells, its expression in the exocrine pancreas raises questions as it could be detected or not in the ductal or acinar cells according to the study (Horsch et al., 1997; Xu et al., 1999; Korner et al., 2007; Tornehave et al., 2008; Gier et al., 2012a). Importantly, inflammation and/or tissue damage can promote neoplasia by altering the fate of acinar and endocrine differentiated cells which can transdifferentiate to ductal cells, thus leading to ductal cell proliferation and preneoplastic lesion formation eventually progressing to pancreatic cancer (Jura et al., 2005; Means et al., 2005; Hernandez-Munoz et al., 2008; Gidekel Friedlander et al., 2009; Logsdon and Ji, 2009; Rebours et al., 2009; Perez-Mancera et al., 2012). Other studies carried on normal and diabetic mice and rats treated with exenatide or liraglutide with or without induction of experimental pancreatic injury did not find any relationship between incretin therapy and the development of pancreatic disease such as pancreatitis and pancreatic tumor (Koehler and Drucker, 2006; Koehler et al., 2009; Tatarkiewicz et al., 2010). But, in these last studies, GLP-1 agonists administration did not exceed 6 days or 4 weeks. Nevertheless, exenatide treatment upregulated PAP/Reg3b (pancreatitis-associated protein) expression as already observed in the course of pancreatic carcinogenesis and pancreatitis (Graf et al., 2006; Gigoux et al., 2008; Koehler et al., 2009; Tatarkiewicz et al., 2010). At last, Nyborg et al. (2012) did not observe pancreatitis in non-diabetic mice, rats, or monkeys after 2 years of liraglutide treatment at exposure levels up to 60 times higher than in humans.

There are very few data on GLP-1R-induced proliferative signaling in pancreatic duct cells. Gier et al. (2012a) showed that exenatide induced proliferative signaling pathways in human pancreatic duct cell line by increasing CREB and ERK1/2 phosphorylation and cyclin D1 expression. ERK1/2 phosphorylation induced by exenatide is dependent of EGF-R activation (Buteau et al., 2003; MacDonald et al., 2003). Koehler and Drucker (2006) also showed that exenatide increased cAMP or induced ERK1/2 activation in some pancreatic cancer cell lines although the proliferation of these cell lines was not modulated.

#### The thyroid

Elashoff et al. (2011) showed a 4.7-fold increase in the incidence of thyroid cancer in diabetic patients treated with exenatide as compared to other therapies (rosiglitazone, nateglinide, repaglinide, and glipizide), by analyzing the U.S. FDA’s database of reported adverse events. In contrast, Hegedus et al. (2011) reported no significant risk for the activation or growth of C-cell cancer in response to liraglutide over a 2-year period. Nevertheless, GLP-1R expression was found in thyroid glands of 20, 91, and 100% of patients with papillary carcinoma, medullary thyroid cancer (MTC), and C-Cell hyperplasia, respectively (Gier et al., 2012b). GLP-1R could be also detected in human normal thyroids (Bjerre Knudsen et al., 2010; Gier et al., 2012b). Therefore, GLP-1 analogs might increase the risk of thyroid C-cell pathology, but this awaits confirmation in humans.

Preclinical studies carried out on rodents treated with liraglutide or exenatide showed a higher incidence of C-cell neoplasia and tumor formation in the thyroid [European Medicines Agency (EMA), 2006, 2009, 2011; Bjerre Knudsen et al., 2010; U.S. Food and Drug Administration, 2011; Bulchandani et al., 2012; Madsen et al., 2012; Victoza (Liraglutide) Injection, 2012]. Indeed, a continuous exposure to liraglutide or exenatide was associated with marked increases in plasma calcitonin and in the incidence of C-cell hyperplasia. These effects were mediated by the GLP-1R as they were not seen in GLP-1R knockout mice (Bjerre Knudsen et al., 2010; Madsen et al., 2012). C-cell hyperplasia is considered as a preneoplastic lesion that constitutes *in situ* carcinoma of the thyroid C-cells (LiVolsi, 1997) and calcitonin, an hormone secreted by thyroid C-cells, is regarded as an important clinical biomarker for C-cell diseases such as MTC and hereditary C-cell hyperplasia because of its high sensitivity and specificity (Elisei et al., 2004; Costante et al., 2007; Machens et al., 2009). Neoplasms were not observed in monkeys after long-term liraglutide administration, indicating that GLP-1 induced C-cell proliferation in rodents but not in primates and suggesting that possible species-specific differences in GLP-1R expression and activation might occur in the thyroid (Bjerre Knudsen et al., 2010).

There are very few data on GLP-1R-induced proliferative signaling in thyroid C-cells. Chronic administration of liraglutide did not modify ERK phosphorylation, but increased ribosomal S6 phosphorylation, a downstream target of mTor and PI3K activation which plays a role in regulating cell proliferation and survival by growth factors (Sengupta et al., 2010; Madsen et al., 2012).

In conclusion, results obtained from preclinical and clinical studies tend to support a pro-tumoral action of GLP-1 in the pancreas and the thyroid, although few studies contradict this role. The relatively short time of chronic treatment with GLP-1 analogs in some studies could explain the absence of significative pro-tumoral effects. Moreover, this raises the question of whether GLP-1 can induce preneoplastic lesions and cancer alone or enable pre-existing lesions to progress to cancer. Further studies should be conducted to determine whether GLP-1 agonists induce or sensitize to pancreatic and thyroid diseases, by comparing chronic administration of GLP-1 mimetics in rodents presenting or not previous injury in the pancreas and the thyroid. However, it is important to note that diabetes is recognized to increase the incidence of pancreatitis and of a variety of cancers, including breast, pancreas, and colon cancers (Giovannucci et al., 2010; Girman et al., 2010; Pandey et al., 2011). Of note, GLP-1R is overexpressed in neuroendocrine pancreatic tumors, more particularly in insulinomas (Korner et al., 2007; Christ et al., 2010). In the current state of knowledge, GLP-1 agonists remain contra-indicated in patients with a personal or family history of MTC or multiple endocrine neoplasia type-2 (Anonymous, 2010; Victoza (Liraglutide) Injection, 2012). Importantly, Risk Evaluation and Mitigation Strategies program including a FDA safety warning published recommendations regarding the risk of thyroid cancer and pancreatitis after use of liraglutide and after dose increases (U.S. Food and Drug Administration, 2011).

Very few data are available on the proliferative intracellular pathways mediated by GLP-1R in pancreatic ductal cells and thyroid C-cells. Nevertheless, in the current state of knowledge, GLP-1R induces proliferation of these cells by same intracellular pathways as in the pancreatic β-cells. The identification of GLP-1 analogs that promote insulin secretion to treat type-2 diabetes without inducing pro-tumoral effects is therefore a timely challenging issue. Glucose-insulinotropic peptide (GIP) incretin could be also another alternative in type-2 diabetes treatment, especially as the GIP receptor (GIP-R) was not expressed in the normal thyroid and the exocrine pancreas unlike GLP-1R (Ahren, 2009; Waser et al., 2011, 2012). But, these clinical indication of GIP should be effective only after normalization of patient’s glycemia which can restore the expression of GIP-R in β-cells (Holst et al., 1997; Vilsboll et al., 2002; Piteau et al., 2007; Younan and Rashed, 2007).

## The Estrogen Receptors ER/GPER

**Figure 3:**
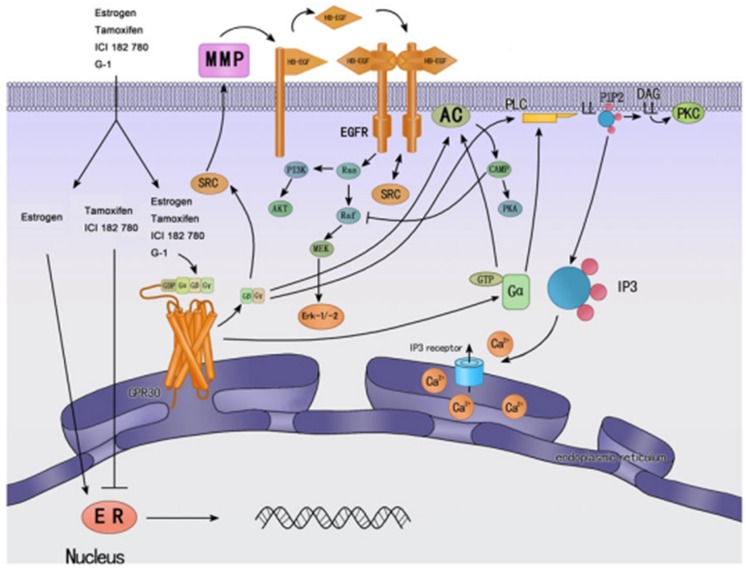
**Cellular signaling mechanisms of GPER and classic nuclear estrogen receptors ERs (↓ activate; ┴ inhibit)**. ERs are widely accepted as mainly mediating gene transcriptional regulation. Tamoxifen is an ER antagonist in some tissue, such as breast cancer, while has agonistic effects in other tissues, such as endometrium. GPER was found predominantly in the endoplasmic reticulum; estrogen and tamoxifen can bind GPER, and then activate multiple cellular effectors, such as ERK, PI3K, and PLC, and other rapid cellular processes. Most of them are mediated by transactivation of EGF-R. Reprinted from Endocrinology (Wang et al., 2010).

Estrogen hormone regulates the growth and the differentiation of many tissues playing a critical role in the development of the reproductive system but also in the nervous, immune, vascular, muscular, skeletal, and endocrine systems. The binding of 17β-estradiol, the natural endogenous estrogen, to the estrogen receptors ERα and ERβ (ER) is the main mechanism responsible for the diverse biological effects of the hormone (Pedram et al., 2006; Meyer and Barton, 2009; Meyer et al., 2009). These highly homologous receptors can shuttle between the cytoplasm and the nucleus and function as ligand-activated nuclear transcription factors that bind *cis*-acting estrogen response elements in the promoter and enhancer regions of hormonally regulated genes (genomic effects of estrogen) (Figure 3) (Ring and Dowsett, 2004; Edwards, 2005; Carroll and Brown, 2006). Estrogen also induces some rapid biochemical responses to estrogen stimulation which occur in seconds to minutes, such as the increase in intracellular free calcium and the activation of multiple intracellular kinases including ERK, PI3K, PKA, and PKC (non-genomic effects of estrogen) (Chen et al., 2008).

Estrogen is the one of the risk factors for breast tumors, which accounts for 40% of cancer among the women and approximately 50% of all breast cancers demonstrated elevated levels of ER expression (Pike et al., 2004). Consequently, anti-estrogen therapy has been extended such as the gold standard tamoxifen (Figure 3) (Deroo and Korach, 2006; Lorand et al., 2010). Unfortunately, long-term treatment with tamoxifen is associated with adverse effects such as an increased incidence of endometrial cancer and with breast cancer resistance to hormonotherapy. Moreover, these events were shown to be associated with G protein signaling- or growth factor-mediated pathways which were not blocked by tamoxifen antagonist, leading to the prediction that an alternative membrane-bound estrogen receptor exists (Wehling, 1997; Hammes and Levin, 2007; Meyer and Barton, 2009). In fact, an orphan GPCR was identified as an estrogen-binding membrane GPCR from vascular and cancer cells and is now included in the official GPCR nomenclature and was designated GPER or GPR30 by the International Union of Pharmacology (Revankar et al., 2005; Thomas et al., 2005; Prossnitz et al., 2008a; Alexander et al., 2011). Its localization seems to be predominantly intracellular due to the constitutive internalization of plasma membrane GPER (Figure 3) (Revankar et al., 2005; Otto et al., 2008; Cheng et al., 2011; Sanden et al., 2011). GPER is widely expressed in cancer cell lines and primary tumors of the breast (Carmeci et al., 1997; Filardo et al., 2000; Revankar et al., 2005; Albanito et al., 2008a), endometrium (Vivacqua et al., 2006a; Leblanc et al., 2007; He et al., 2009), ovaries (Albanito et al., 2007, 2008b; Henic et al., 2009), thyroid (Vivacqua et al., 2006b), lung (Siegfried et al., 2009), prostate (Chan et al., 2010), and testicular germ cells (Franco et al., 2011). GPER does not only bind estrogens but also other substances such as tamoxifen which displays estrogenic agonist activity on GPER notably in the reproductive systems (Figure 3) (McDonnell, 1999; Filardo et al., 2000; Thomas and Dong, 2006; Jordan, 2007; Albanito et al., 2008b; Orlando et al., 2010; Chevalier et al., 2012). Indeed, tamoxifen stimulates the cell proliferation and growth of cell lines of thyroid, ovarian, endometrial, and breast cancers (Filardo et al., 2000; Thomas et al., 2005; Vivacqua et al., 2006a; Albanito et al., 2007; Prossnitz et al., 2008b; Pandey et al., 2009). The discovery of GPER-selective agents and the elaboration of GPER knockout mice helped to examine GPER signaling pathways and strongly supported that GPER is associated with cancer proliferation, migration, invasion, metastasis, differentiation, prognosis, and drug resistance (Prossnitz et al., 2008b; Wang et al., 2010).

### Could estrogen antagonists used in breast cancer treatment induce cancer in other tissues?

An increased incidence of uterine malignancies in association with tamoxifen treatment has been reported. The incidence and severity of endometrial cancer increased by 4- to 6.9-fold in women with 5 years of exposure to tamoxifen (van Leeuwen et al., 1994; Bernstein et al., 1999; Bergman et al., 2000; Goldstein, 2001). Uterine sarcoma has been also reported to occur more frequently among long-term users (≥2 years) of tamoxifen than non-users (Wickerham et al., 2002). In support to these data, tamoxifen has been shown to stimulate the proliferation and the invasion of uterine cells *in vivo* and of human endometrial carcinoma cell lines and these effects were mediated by GPER (Gottardis et al., 1988; Jamil et al., 1991; Schwartz et al., 1997; Du et al., 2012a). Indeed, tamoxifen promoted cell proliferation and invasion of the human endometrial cancer cell lines ISHIKAWA and KLE, while the down-regulation of GPER partly or completely prevented these effects (Du et al., 2012a). GPER is widely expressed in primary tumors of endometrium including ER-negative endometrial carcinomas (He et al., 2009). High levels of GPER expression correlate with an increased incidence of endometrial cancer and with tamoxifen-induced uterine pathology and predict poor survival in endometrial cancer (Smith et al., 2007; Ignatov et al., 2010a). All together, these data strongly support that tamoxifen treatment might have a cancer-promoting effect through GPER.

G protein-coupled estrogen receptor promotes carcinogenesis by endometrial cancer cells as down-regulation of GPER led to reduce growth and invasion by RL95 endometrial cancer cells treated with 17β-estradiol and to decrease tumorigenesis *in vivo* (He et al., 2009, 2012). GPER mediates the proliferative effects of estrogen and tamoxifen in endometrial cancer cells through EGF-R transactivation leading to the activation of ERKs and PI3K pathways (Vivacqua et al., 2006a; Prossnitz et al., 2008b; He et al., 2009, 2012; Du et al., 2012a; Lappano et al., 2012; Wei et al., 2012). GPER also mediates invasion by endometrial cancer cells through the stimulation of ERK pathway, as well as the increase of interleukin-6 secretion, leading to the production and activation of matrix metalloproteinases MMP-2 and MMP-9 known to degrade extracellular matrix components and to be involved in cancer invasion and metastasis (He et al., 2009, 2012; Du et al., 2012a).

### Could GPER be involved in breast cancer resistance to hormonotherapy?

The majority of breast cancers is ER-positive and depends on estrogen for growth. Therefore, blocking estrogen signaling remains the strategy of choice for the treatment and the prevention of breast cancer. Tamoxifen is the prototypical drug that targets ER. It presents potent anti-estrogenic properties and has been used extensively for the past 40 years to treat and prevent breast cancer (Jordan and Morrow, 1999). Tamoxifen treatment is very effective in tumors expressing ER receptors and significantly reduces the mortality of breast cancer patients (Jordan and Morrow, 1999; Powles et al., 2007). Many patients with ER-positive breast cancer have benefited from anti-hormonal treatment, but unfortunately, almost 30–50% of patients with advanced disease did not respond to first-line treatment with tamoxifen. Furthermore, long-term tamoxifen therapy causes the development of acquired resistance (Early Breast Cancer Trialists’ Collaborative Group (EBCTCG), 2005). Indeed, development of resistance is very frequent and tamoxifen is not effective for more than 5 years (Saphner et al., 1996; Clarke et al., 2001; Early Breast Cancer Trialists’ Collaborative Group (EBCTCG), 2005; Barron et al., 2007; Brewster et al., 2008).

The tumor resistance to tamoxifen treatment is associated to a decrease or a loss of ER expression and to an increase of GPER expression. GPER protein is expressed in ∼50% of all breast cancers including half of ER-negative tumors and correlates with increased tumor size and metastasis (Filardo et al., 2006; Ignatov et al., 2011). Moreover, GPER protein expression is increased in breast tumors of patients treated only with tamoxifen and in tamoxifen resistant tumor tissues correlating with a poor relapse-free survival in patients treated with tamoxifen (Filardo et al., 2006; Ignatov et al., 2011). *In vitro* prolonged tamoxifen treatment leads to an increased cell surface expression of GPER and also to clonal selection of GPER-positive MCF-7 breast cancer cells (Ignatov et al., 2010b). Thus, GPER expression is associated with an increased risk of resistance to tamoxifen and patients with breast cancer who have high GPER protein expression should not be treated with tamoxifen alone.

G protein-coupled estrogen receptor mediates the proliferative and tamoxifen-resistance effects through EGF-R transactivation leading to the phosphorylation of ERK and Akt (Filardo et al., 2000; Prossnitz et al., 2008b; Ignatov et al., 2010a,b). Thus, ERK and Akt can further stimulate transcription of different genes (even ER), leading to cell proliferation, and interfere with the activation of Smad proteins, known effectors of the TGF-β signaling, an important intracellular pathway involved in the inhibition of tumor progression (Clarke et al., 2001; Kleuser et al., 2008; Yoo et al., 2008; Ignatov et al., 2010b).

In conclusion, tamoxifen has been the only available hormonal option for the systemic treatment for breast cancer from 1973 to 2000. Despite the clinical success of tamoxifen, the development of drug resistance and endometrial cancers leads to the requirement of alternative hormonal therapy. In this regard, the knowledge of the contribution of GPER-mediated signaling in the undesired effects of estrogenic antagonist uses for breast cancer treatment should allow the future development of new molecules. Moreover, further researches are required to define the role of GPER signaling in estrogen undesired physiological effects and to elucidate the role of non-selective estrogen receptor ligands in health and disease.

## New Hopes to Overcome Undesired Effects

G protein-coupled receptors are generally expressed in several different tissues and involved in numerous physiological processes. Many natural ligands can bind and activate several subtypes of GPCR. This is illustrated, for example, with cholecystokinin and somatostatin receptors (Guillermet-Guibert et al., 2005; Dufresne et al., 2006). Ligands can also activate different classes of receptors as illustrated before with estrogen (Prossnitz et al., 2008a). Such a diversity of receptors activation following agonist administration can engage multiple intracellular signaling pathways and be responsible for adverse effects in treated patients. Furthermore, biological effects triggered by the same GPCR result from the activation of G protein-dependent and -independent intracellular signaling pathways. Recently, signaling engaged after GPCR recruitment of β-arrestin proteins have emerged as new G protein-independent intracellular signaling pathways (Luttrell and Gesty-Palmer, 2010; Rajagopal et al., 2010a). To increase the complexity, a single GPCR has pleiotropic signaling properties and each signal can crosstalk at different levels with the transactivation of cell surface receptor having tyrosine kinase activity (EGF-R, PDGF-R, FGF-R, for examples) or serine/threonine kinase activity (TGF-β, for example) or with the formation of multimers, thus potentially influencing the signaling pathways of the different receptors (Burch et al., 2012; Wang and Lewis, 2013). Indeed, numerous biochemical and biophysical studies supports that GPCRs can form physiologically relevant homo-, hetero-, or oligo-mers (Angers et al., 2002). Homodimerization of the GLP-1R was shown to be critical for selective coupling of the receptor to physiologically relevant signaling pathways (Harikumar et al., 2012). Indeed, disruption of GLP-1R homodimerization completely abrogated the intracellular calcium mobilization response whereas it slightly reduced cAMP formation and phosphorylation of ERK. Furthermore, GLP-1R dimerization can discriminate between peptide and non-peptide-mediated receptor activation. In the chemokine receptors family, antibodies against the CCR2b promoted the receptor dimerization and second messenger production (Rodriguez-Frade et al., 1999), whereas an antibody directed against CCR5, that induces receptor dimerization, inhibits its function (Vila-Coro et al., 2000). Many studies was also conducted to analyze the role of GPCR heterodimerization and supported that heterodimerization could be the source of additional pharmacological properties which are different from those of the individual receptors. As a first example, the co-expression of the δ- and κ-opioid receptors in the same cell leads to an almost complete loss of binding to selective δ- and κ-ligands while preserving binding to non-selective ligands (Jordan and Devi, 1999). As a second example, somatostatin receptor SSTR1 displays internalization in cells when it is co-expressed with SSTR5, whereas monomeric SSTR1 is resistant to internalization in contrast to monomeric SSTR5, suggesting that the SSTR1 trafficking is modified by its heterodimerization with SSTR5 (Rocheville et al., 2000). Thus, homo- and heterodimerization between GPCR cause complexity in the receptor pharmacological properties that can be responsible of synergistic or antagonistic signaling cross-talks. This GPCR pharmacological and signaling complexity could account for unexpected pharmacological effects and have dramatic impacts on drug development. All together, these hallmarks indicate that undesired adverse effects can be expected with a prolonged agonist administration that targets a GPCR (Table 1). Moreover, many GPCR have already been shown to present proliferative and pro-tumoral properties (Table 2), suggesting that an increase of preneoplastic lesions and cancer incidence can potentially occur following chronic activation of GPCR. Thus, deciphering which signaling networks are engaged and orchestrated following GPCR receptor activation appears to be primordial to unveil their contribution in the cell fate.

**Table 2:** **Examples of Neuropeptide GPCRs with pro-tumoral activities and properties**.

Ligands	Receptors	Target	Activity	Reference
Adrenocorticotropic hormone	MC2R	Prostate	Proliferation	Hafiz et al. (2012)
Angiotensin II	AT1R	Breast	Proliferation, adhesion, migration, invasion	Rodrigues-Ferreira et al. (2012), Du et al. (2012b)
Gastrin	CCK2	Pancreas, stomach	Proliferation, adhesion	Dufresne et al. (2006), Cayrol et al. (2006), Bierkamp et al. (2004), Clerc et al. (2002a,b), Mathieu et al. (2005)
Glucagon-like peptide-1	GLP-1R	Exocine pancreas Thyroid	Pro-tumoral	Nachnani et al. (2010), Gier et al. (2012a), Elashoff et al. (2011), Bjerre Knudsen et al. (2010), Madsen et al. (2012), Victoza (Liraglutide) Injection (2012)
Ghrelin	GHS-R	Breast	Proliferation, migration	Jeffery et al. (2005)
		Prostate	Proliferation	Yeh et al. (2005), Jeffery et al. (2002)
		Endometrium	Proliferation	Fung et al. (2010)
		Stomach		Tian and Fan (2012)
Melanin-concentrating hormone	MCHR1	colon	Pro-tumoral, apoptosis	Nagel et al. (2012)
Neuromedin B	NMB-R	Colon	Proliferation	Matusiak et al. (2005)
		Breast	Apoptosis, proliferation	Park et al. (2011)
Neuromedin U	NMU-R2	Pancreas	Migration, invasion	Ketterer et al. (2009)
Neuropeptide Y	NPY Y5-R	Breast	Proliferation, migration	Medeiros et al. (2012), Sheriff et al. (2010)
	NPY Y2-R	Neuroblastoma	Proliferation, angiogenesis	Lu et al. (2010)
	NPY Y1-R	Prostate	Proliferation	Ruscica et al. (2006)
Neurotensin	NTSR1/3	Colon	Proliferation, Pro-tumoral	Muller et al. (2011)
Parathyroid hormone	PTH-R	bone	Pro-tumoral	Subbiah et al. (2010), Hodsman et al. (2005)
Pituitary adenylate cyclase-activating polypeptide	PAC1	Lung	Proliferation	Moody et al. (2012)
Prokineticin 1/2	PROKR1/2	Thyroid	Angiogenesis	Monnier and Samson (2010)
Relaxin	RXFP1	Uterus	Proliferation, apoptosis	Suzuki et al. (2012)
		Prostate	Pro-tumoral, metastasis, proliferation	Feng et al. (2010)
Urotensin II	UTR	Prostate	Migration, invasion	Grieco et al. (2011)
		Lung	Proliferation	Wu et al. (2010)
Vasoactive intestinal peptide	VPAC1	Prostate	Proliferation, migration	Fernandez-Martinez et al. (2010)
		Brain (glioblastoma)	Migration	Cochaud et al. (2010)
		Breast	Angiogenesis	Valdehita et al. (2012)
		Lung	Proliferation	Moody et al. (2000)
26RFa	GPR103	Prostate	Migration	Alonzeau et al. (2012)

One strategy to overcome these limitations would be to examine the initial steps following receptor activation. The release of X-ray structures of agonist/GPCR complexes (Chung et al., 2011; Lebon et al., 2011; Warne et al., 2011; Xu et al., 2011; Audet and Bouvier, 2012), the numerous biophysical and biochemical studies (Granier et al., 2007; Kahsai et al., 2011; Liu et al., 2012; Rahmeh et al., 2012) have enabled to show that different and selective ligands, named biased ligands, can induce or stabilize distinct receptor conformations and activate one (or several) signaling pathway(s) in contrast to non-biased agonists which activate all the signaling pathways (Vaidehi and Kenakin, 2010). Thus an understanding of the structure and dynamics of the ensemble of receptor conformations would greatly help the design of small molecules with functional selectivity or “biased signaling” properties and would provide more specific and efficient new drugs. Receptor structure/activity relationship studies, structure- and docking-based virtual screening are now widely applied in drug discovery and must take in account the existence of different receptor conformations activating specific signaling pathways.

Although GPCRs can modulate a large variety of distinct signaling pathways, classification of biased ligands are actually restricted to two groups depending on their ability to activate two main transduction pathways (Whalen et al., 2011): (1) G protein-biased ligands which promote G protein activation without β-arrestin recruitment and (2) β-arrestin-biased ligands which recruit β-arrestin to the receptor and initiate consecutive signaling pathways in the absence of G protein activation.

The vast majority of biased ligands identified so far exhibits exclusive β-arrestin activity for a number of receptors (Rajagopal et al., 2010a; Whalen et al., 2011), including the AT1 angiotensin II receptor, β1- and β2-adrenergic receptors, or the CXCR7 decoy receptor (Wei et al., 2003; Wisler et al., 2007; Kim et al., 2008; Rajagopal et al., 2010b). The parathyroid hormone (PTH) analog, D-Trp(12),Tyr(34)-PTH(7-34), binds the PTH receptor 1 (PTHR1) and activates β-arrestin-dependent but not classical G protein-dependent signaling (Gesty-Palmer et al., 2009; Gesty-Palmer and Luttrell, 2011). In mice, this PTH biased agonist induces anabolic bone formation without stimulating bone resorption, comparatively with the non-selective agonist PTH(1-34) which induces both functions. Thus, this PTHR1 biased ligand may present interesting properties for the treatment of metabolic bone diseases such as osteoporosis and is a proof of concept that the exploitation of β-arrestin biased agonism may offer therapeutic benefit.

Few ligands have been yet identified as perfect G protein-biased ligands, namely inducing G protein signal transduction without any β-arrestin recruitment (Whalen et al., 2011). GMME1 ligand binding to the CCR2 chemokine receptor leads to calcium mobilization, caspase-3 activation and consecutive cell death, but does not recruit β-arrestin2 (Rafei et al., 2009). Selective ligands that activate G protein-coupling by FSH-R (follicle-stimulating hormone receptor) and PTH-1R have been also reported (Bisello et al., 2002; Wehbi et al., 2010). Of note, some ligands classified as G protein-biased can induce a weak β-arrestin recruitment by the targeted GPCR (Whalen et al., 2011). For example, oxyntomodulin and glucagon are full agonists in GLP-1R-mediated cAMP accumulation but partial agonists in recruiting β-arrestins to this receptor, suggesting that oxyntomodulin and glucagon are biased ligands on the GLP-1R (Jorgensen et al., 2007).

Interestingly, some ligands are biased in regard to the different G protein families and can trigger opposite cellular responses (Reversi et al., 2005; Sensken et al., 2008). For example, oxytocin receptors (OTR) coupling to Gi inhibits cell proliferation, whereas its coupling to Gq stimulates cell proliferation. Atosiban, an oxytocin derivative, was shown to act as a competitive antagonist on OTR/Gq coupling, and to display agonistic properties on OTR/Gi-coupling, thereby leading to the selective inhibition of cell growth (Reversi et al., 2005; Busnelli et al., 2012). SOM230 which activates the somatostatin receptor sst2A behaves as agonist for Gi coupling and inhibition of adenylyl cyclase, but antagonizes somatostatin’s actions on intracellular calcium and ERK phosphorylation which can be activated by a Gi/Go independent process (Cescato et al., 2010).

Biased signaling can also exist with respect to other signaling proteins than G proteins and arrestins. The internalization of apelin receptor takes different signaling pathways depending of the apelin isoforms (Lee et al., 2010). Indeed, apelin-13-activated receptors dissociated rapidly from β-arrestin1 and were recycled to the cell surface through a Rab4-dependent mechanism, while the apelin-36-internalized receptors trafficked with β-arrestin1 to intracellular compartments and were targeted by Rab7 to lysosomes for degradation. CCL19 and CCL21 ligands both induce β-arrestin2 recruitment by the receptor CCR7, but activate different GRK (G protein receptor kinase) isoforms (Zidar et al., 2009). Indeed, CCL19 leads to robust CCR7 phosphorylation and β-arrestin2 recruitment catalyzed by both GRK3 and GRK6 whereas CCL21 activates GRK6 alone. The functional consequences are that only CCL19 leads to classical receptor desensitization whereas both agonists are capable of signaling through GRK6 and β-arrestin2 to ERK kinases.

### The GLP-1R

Results obtained from preclinical and clinical studies tend to support a pro-tumoral action of GLP-1 in the pancreas and the thyroid. Deciphering the signaling networks engaged following GLP-1R agonist administration in pancreatic ductal cells and thyroid C-cells comparatively to pancreatic β-cells is critical to unveil their contribution in the different cellular processes. The identification of GLP-1 analogs that promote insulin secretion to treat type-2 diabetes without inducing pro-tumoral effects is therefore a timely challenging issue. Like most GPCRs, the GLP-1R couples to different classes of heterotrimeric G proteins, including Gαs, Gαq, and Gαi, regulatory proteins such as the β-arrestins, and activates multiple signaling pathways such as cAMP production, intracellular calcium mobilization, phosphorylation of ERK1/2. While GLP-1 can activate all of these signaling pathways, some compounds were shown to present biased activity on GLP-1R. Oxyntomodulin and glucagon biased the GLP-1R toward cAMP accumulation over the recruitment of β-arrestins, BMS21 compound toward ERK1/2 activation and cAMP production over β-arrestins recruitment, BETP compound toward calcium mobilization and β-arrestins recruitment over cAMP production and ERK1/2 activation (Jorgensen et al., 2007; Wootten et al., 2013). Moreover, some molecules acting as allosteric modulators were shown to modulate GLP-1R agonist-mediated signaling pathways (Willard et al., 2012; Wootten et al., 2013). For example, BETP increases the affinity of GLP-1R to oxyntomodulin and potentiates the activation of cAMP production induced by oxyntomodulin.

The most crucial GLP-1R signaling pathway for enhancing glucose-dependent insulin secretion involved the receptor coupling to Gαs proteins and the activation of cAMP production (Baggio and Drucker, 2007). While GLP-1 analogs are currently tested for their capacity to activate Gαs protein and cAMP production, their effects on other signaling pathways, particularly those involved in cell proliferation, should be included. Moreover, the analysis of pharmacological ligand properties should be done on the main cellular target, the pancreatic β-cell, but also on cells involved in carcinogenic side effects. This could enable the design and development of improved therapeutics that have the ability to fine-tune receptor signaling leading to beneficial therapeutic outcomes while reducing side effect profiles. The use of allosteric ligands in addition to GLP-1R biased agonists could also provide a therapeutic advantage to target a specific receptor response toward signaling pathways promoting insulin secretion over cell proliferation.

### The GPER

Despite the clinical success of tamoxifen in breast cancer treatment, the development of drug resistance and endometrial cancers involving the GPER leads to the requirement of alternative hormonal therapy. In this regard, the contribution of GPER-mediated responses estrogen antagonists must be considered in the future development of anti-estrogenic molecules. Recent studies based on pharmacological structure/function relationship on ER and/or GPER have identified selective GPER antagonists which completely block uterine epithelial cell proliferation mediated by GPER and which are poorly active or inactive on ER (Dennis et al., 2009, 2011; Burai et al., 2012). Future studies utilizing GPER-selective ligands will further define the role of this receptor *in vivo* and open the door to the generation of diagnostics and therapeutics directed at individual or both estrogen receptors. Such compounds might represent an important new approach for cancer therapy, thus increasing the armamentarium of drugs used to treat estrogen-sensitive and resistant cancers. On the other hand, aromatase inhibitors which act by preventing the enzyme aromatase to convert androgens into estrogen have been also brought forward as a potential alternative (Josefsson and Leinster, 2010; Abdulkareem and Zurmi, 2012).

In conclusion, GPCRs provide huge therapeutic opportunities; some are already in use. The progress in the knowledges of signaling pathways downstream of these receptors and the effects arising, their regulation by pharmacological agents, and the data from the receptor structure provide new opportunities which should lead to new generation of ligands with minimized side effects.

## Conflict of Interest Statement

The authors declare that the research was conducted in the absence of any commercial or financial relationships that could be construed as a potential conflict of interest.
